# Comparative BAC-based mapping in the white-throated sparrow, a novel behavioral genomics model, using interspecies overgo hybridization

**DOI:** 10.1186/1756-0500-4-211

**Published:** 2011-06-21

**Authors:** Michael N Romanov, Jerry B Dodgson, Rusty A Gonser, Elaina M Tuttle

**Affiliations:** 1 Dept. of Biology, Indiana State University, Terre Haute, Indiana 47809, USA; 2 Dept. of Microbiology and Molecular Genetics, Michigan State University, East Lansing, Michigan 48824, USA

## Abstract

**Background:**

The genomics era has produced an arsenal of resources from sequenced organisms allowing researchers to target species that do not have comparable mapping and sequence information. These new "non-model" organisms offer unique opportunities to examine environmental effects on genomic patterns and processes. Here we use comparative mapping as a first step in characterizing the genome organization of a novel animal model, the white-throated sparrow (*Zonotrichia albicollis*), which occurs as white or tan morphs that exhibit alternative behaviors and physiology. Morph is determined by the presence or absence of a complex chromosomal rearrangement. This species is an ideal model for behavioral genomics because the association between genotype and phenotype is absolute, making it possible to identify the genomic bases of phenotypic variation.

**Findings:**

We initiated a genomic study in this species by characterizing the white-throated sparrow BAC library via filter hybridization with overgo probes designed for the chicken, turkey, and zebra finch. Cross-species hybridization resulted in 640 positive sparrow BACs assigned to 77 chicken loci across almost all macro-and microchromosomes, with a focus on the chromosomes associated with morph. Out of 216 overgos, 36% of the probes hybridized successfully, with an average number of 3.0 positive sparrow BACs per overgo.

**Conclusions:**

These data will be utilized for determining chromosomal architecture and for fine-scale mapping of candidate genes associated with phenotypic differences. Our research confirms the utility of interspecies hybridization for developing comparative maps in other non-model organisms.

## Background

Much of our current knowledge of genetics and genomics comes from traditional model organisms that are often raised for many generations in the laboratory. Although model organisms offer several advantages, as with inbred strains where it is often easier to isolate the factors associated with particular traits (e.g. [1]), they can also show altered behaviors, physiologies, and genetic responses due to extended exposures to laboratory environments (e.g. [2-5]). Traits of interest may not be expressed or might be entirely absent from the phenotypic repertoire of a model organism [6]. Finally, in laboratory systems it is difficult to determine the relative influence of genes and environments, which can be absolutely essential considering that many complex traits have low heritabilities, exhibit strong gene-by-environment effects, or are influenced by epigenetics. Studies of "non-model" organisms can therefore advance our understanding of genetic and genomic patterns and processes as these species are still subject to evolutionary forces such as selection, gene flow, and drift [6,7].

For non-model organisms to be useful for genomic inquiry, their genomes need to be structurally and functionally characterized. Genomic tools and resources, initially developed from species determined to be either medically or economically significant, have paved the way for comparative studies of the genomes of non-model organisms. For example, the first avian genome to be sequenced was the chicken (*Gallus gallus*) [8,9]. Since then, several other avian genomes have been sequenced and/or characterized to some extent, including other economically important species such as the turkey (*Meleagris gallopavo*) [10], main neurobiological models such as the zebra finch (*Taeniopygia guttata*) [11], ecologically essential species such as flycatchers (*Ficedula spp*.) [12-15], and species critical to conservation such as the California condor (*Gymnogyps californianus*) [16-18]. Comparative genomics methodologies have illuminated many similarities [14,15,19-21] and differences [11,12,22-24] across these taxonomic groups. Birds occupy a unique evolutionary position and many have been so well studied that continued comparative work within this group promises to remain fruitful and open new avenues for fundamental and applied biological research.

**Figure 1:**
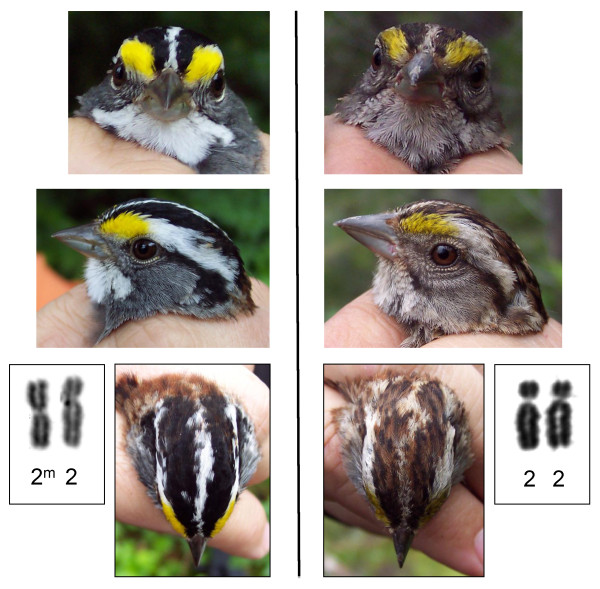
**Various views of the plumage morphs of the white-throated sparrow**. Two morphs are shown: A, the white morph, and B, the tan morph. Morph is absolutely associated with the presence (i.e. ZAL2^m^/ZAL2 = white) or absence (i.e. ZAL2/ZAL2 = tan) of a chromosomal rearrangement.

The white-throated sparrow (*Zonotrichia albicollis*), with its morphological, behavioral and chromosomal polymorphisms, represents a new system to study genomic mechanisms underlying variation [17]. Males and females in this species occur as either white or tan morphs [25] (Figure 1) that exhibit alternate behaviors. White morphs are promiscuous and show lower parental effort, whereas tan morphs are monogamous and exhibit higher levels of parental care [26]. Behavioral and morphological differences in the two morphs appear to have a genetic basis [27,28]: white birds are heterozygous for a complex rearrangement on chromosome 2 (i.e. ZAL2^m^/ZAL2), whereas tan birds do not carry the rearrangement (i.e. ZAL2/ZAL2) [17,29,30]. Homozygous white birds (ZAL2^m^/ZAL2^m^) are rarely found (< 0.06%; Tuttle, unpublished data). Karyotypic evidence also suggests inter-chromosomal linkage with chromosome 3 [17]. White and tan morphs mate disassortatively [25,27,28], maintaining polymorphism in this species and resulting in pair types that differ in the amount of biparental care they provide [31]. The disassortative pair types also differ in other key behavioral and ecological attributes [26,32-34]. Together, these traits make the species not only an ideal model in which to study the genomics of social behavior, but study of the genomics in this species will also advance our understanding of chromosome structure, immunity and disease, language and learning, as well as fertility and reproduction.

The availability of a genomic BAC library for the white-throated sparrow [30] allows physical mapping, followed by refined cytogenetic and linkage mapping. The integration of these maps, coupled with in-depth sequencing of the targeted regions on white-throated sparrow chromosomes, ZAL2, ZAL2^m ^and other areas (e.g. ZAL3 and ZAL3^a^; see [17]) that might be involved in the observed karyotypic variation, could reveal the nature of morph-specific reproductive strategies. As a first step towards dense physical mapping of white-throated sparrow chromosomes, we undertook screening the sparrow BAC library using a cross-species overgo hybridization approach [35]. The chicken [8] and the zebra finch [11] were used as reference genomes. In these two species, chromosome 3 (GGA3 and TGU3) is known to correspond to ZAL2 [29], and ZAL3 might be orthologous to GGA4 and TGU4. Therefore, we derived numerous white-throated sparrow BAC clones specific for almost all chromosomes, with a focus on GGA3 and GGA4 loci, and developed a first-generation BAC-based comparative physical map.

## Materials and methods

### BAC library

The white-throated sparrow is a North American songbird in the order *Passeriformes*, family *Emberizidae*. This species has a total diploid chromosome number of 82, including the sex chromosomes [17]. The BAC library for the white-throated sparrow (CHORI-264; http://bacpac.chori.org/library.php?id=469) was generated at BACPAC Resources, CHORI, Oakland, CA, using DNA from frozen kidney tissue of a single white female [30]. It consists of 196,354 BAC clones spotted onto 11 nylon filters. The average clone insert size is 144 Kb. For the current study, we screened a representative fraction of the library (4/11) using hybridization of the first four filters.

### Overgo hybridization

Cross-species hybridization followed the published procedure [16,17,35,36]. Briefly, library screening involved four-dimensional filter hybridization based on arranging 216 overgos (~40-bp unique probes for a gene or marker) in six virtual plates, each with 6 rows and 6 columns (i.e. 36 probes per plate). The filter hybridizations for the first three dimensions of plates, rows and columns were conducted using the appropriate plate, row and column pools of 36 overgos, so that one probe was added once per dimension of hybridization. Positive BAC clones for a certain overgo probe were those detected at a specific intersection of plate, row and column pools. An additional fourth dimension composed of 6 virtual diagonal pools was employed to ensure the accuracy of the whole hybridization round that included 24 single hybridizations. For each hybridization, a pool of 36 overgos was labeled with ^32^P nucleotides and hybridized to a BAC filter set as previously described. In the present study, all 216 overgos were radiolabeled at once and used in a series of two consecutive hybridizations by plates and rows (6 pools of 36 overgos for each) and, then, by columns and diagonals, thereby reducing the cost and time of overgo labeling, hybridization, and post-hybridization steps.

The probes were a set of 216 pre-existing overgos mainly derived from the chicken sequence (see additional file 1: Overgo Probes for the list of probes and their description). Numbers of overgos that corresponded to chicken chromosome sequences are shown in Table 1. In some cases, we did not have a sufficient number of the available chicken overgos to evenly cover specific chromosomes. To address this issue, we added 19 turkey EST-derived probes, mostly for GGA3, that were available from the turkey genome project (Dodgson, unpublished data), as well as three GGAZ overgos previously designed using zebra finch ESTs [35].

**Table 1:** Coverage of the chicken genome with 216 overgos selected for the sparrow BAC library screen

Chromosome or linkage group	Chromosome length, Mb	No. of overgos	Average overgo interval, Mb
Macrochromosomes			
GGA1	200.99	21	9.14
GGA2	154.87	12	11.91
GGA3	113.66	98	1.15
GGA4	94.23	12	7.25
GGA5	62.24	4	12.45
Intermediate chromosomes			
GGA6	37.40	4	7.48
GGA7	38.38	4	7.68
GGA8	30.67	4	6.13
GGA9	25.55	3	6.39
GGA10	22.56	3	5.64
Microchromosomes			
GGA11	21.93	3	5.48
GGA12	20.54	3	5.14
GGA13	18.91	3	4.74
GGA14	15.82	2	5.27
GGA15	12.97	2	4.32
GGA16	0.43	4^a^	0.09
GGA17	11.18	2	3.73
GGA18	10.93	2	3.64
GGA19	9.94	2	3.31
GGA20	13.99	2	4.66
GGA21	6.96	2	2.32
GGA22	3.94	2	1.31
GGA23	6.04	2	2.01
GGA24	6.40	2	2.13
GGA25	2.03	2	0.68
GGA26	5.10	2	1.70
GGA27	4.84	2	1.61
GGA28	4.51	2	1.50
LGE22C19W28_E50C23	0.90	2	0.30
Sex chromosomes			
GGAW	0.26	1	0.13
GGAW_random^b^	0.73	1	0.37
GGAZ	74.60	6	10.66
GGAUn_random	63.87	1^a^	31.94

^a^One of the probes selected for GGA16 matches a sequence that has not been assigned yet to a particular chromosome and is designated as 'GGAUn_random' in the chicken genome sequence (Build 2.1).

^b^GGAW_random is a random sequence known to be located on the W chromosome but is yet to be assembled with the rest of the W chromosome sequence.

### Filter image analysis

**Figure 2:**
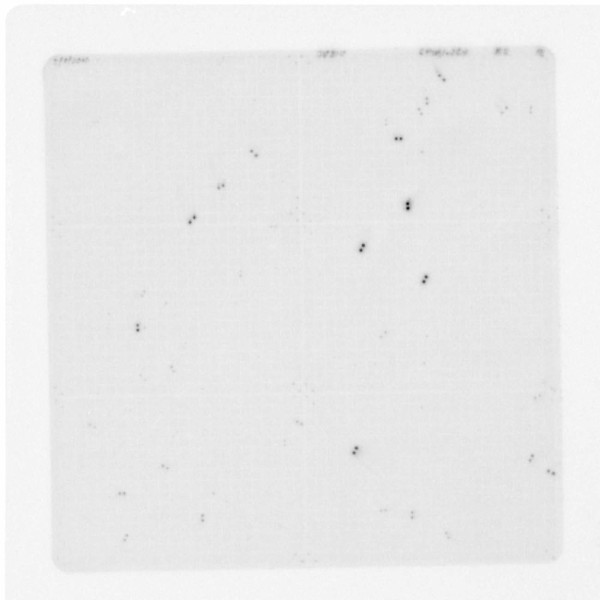
**Image of a sparrow BAC filter hybridized to a set of 36 overgo probes**. The probes were mostly derived from the chicken sequence. The filter has six panels, with gray double spots of anchor probes shown at panel intersections and utilized for filter grid alignment at the computer image analysis stage. Other black and gray double spots represent hybridization signals.

Filters images were obtained with storage phosphor screen scanning using a Typhoon imager (GE Healthcare) (Figure 2). Images were analyzed using ImageQuant (GE Healthcare) and HDFR (Incogen) software packages. Clones positive for at least three of four hybridizations were identified using an in house Microsoft Access program and were examined manually to eliminate spurious identifications.

### Comparative map design

The obtained BAC-gene (BAC-marker) assignments were entered in the final spreadsheet (see additional file 1: Overgo Probes for genes/markers with positive BAC clone information) and served as data points for constructing a BAC-based chicken-sparrow comparative map using the MapChart version 2.2 software [37]. Positions of BAC-gene assignments on the chicken genome sequence (Build 2.1) were retrieved by BLAT/BLAST search using overgo sequences and two genome browsers, Ensembl http://uswest.ensembl.org/Gallus_gallus/Info/Index and NCBI http://www.ncbi.nlm.nih.gov/genome/seq/BlastGen/BlastGen.cgi?taxid=9031. We also identified the same loci on the zebra finch sequence (Build 1.0; Warren et al., 2010).

## Results

### Overgo-based BAC library screen

The estimated length of the CHORI-264 BAC library is 28,274,976 Kb. Since the genome size (C-value) of the white-throated sparrow is approximately 1.37 pg [38], or 1,339,860 Kb, our estimate for the library coverage of the sparrow genome was 21.1X. The four filter subset chosen for the library screening therefore represented a 7.7-fold coverage of the sparrow genome, sufficient to ensure a high success rate of positive BAC clone identification via overgo hybridization.

Almost half of the selected probes (N = 98; Table 1) matched loci (genes and markers) on chicken chromosome 3 (GGA3) that was suggested to be orthologous to ZAL2 and ZAL2^m^, for which we sought a much denser coverage. These probes were evenly spread over the entire GGA3, with an average spacing of 1.15 Mb. Overgo distribution on other chromosomes was less dense, with a mean overgo span for macrochromosomes GGA1 through GGA5 being 7.3 to 12.5 Mb per overgo. For intermediate chromosomes GGA6 through GGA10, it was 5.6 to 7.5 Mb, for michrochromosomes GGA11 through GGA28 it was 0.09 to 5.5 Mb, and for the Z sex chromosome it was 10.7 Mb. Overall chromosome coverage with the 216 chosen overgos was 1033.5 Mb of the total length of 33 chicken autosomes and sex chromosomes, with an average interval of 4.2 Mb.

In the course of the first screen based on 216 probes, we identified 640 positive white-throated sparrow BACs that were assigned to 77 chicken loci, indicating that 35.6% overgos resulted in successful hybridization. The number of detected positive sparrow BACs varied from 0 to 30, with an average being 3.0 clones per overgo. For the 77 successful probes, 8.3 clones per overgo were positive, close to the expected redundancy of the chosen BAC library fraction used for the screening (7.7X). The success rate of overgo hybridization ranged across almost all chromosomes screened between 23.5 and 100% (Table 2).

**Table 2:** Results of the first round of the white-throated sparrow BAC library screen

Chromosome or linkage group	No. of overgos used	No. of successful overgos	Success rate, %	No. of positive BACs	Positive BACs per probe
Macrochromosomes					
GGA1	21	8	38.1	58	7.3
GGA2	12	5	41.7	29	5.8
GGA3	98	23	23.5	192	8.3
GGA4	12	6	50	77	12.8
GGA5	4	4	100	34	8.5
Intermediate chromosomes					
GGA6	4	1	25	8	8
GGA7	4	3	75	25	8.3
GGA8	4	0	0	0	0
GGA9	3	0	0	0	0
GGA10	3	1	33.3	7	7
Microchromosomes					
GGA11	3	3	100	36	12
GGA12	3	2	66.7	8	4
GGA13	3	1	33.3	6	6
GGA14	2	0	0	0	0
GGA15	2	2	100	17	8.5
GGA16	4	1^a^	25	18	18
GGA17	2	2	100	14	7
GGA18	2	1	50	18	18
GGA19	2	1	50	12	12
GGA20	2	1	50	7	7
GGA21	2	1	50	7	7
GGA22	2	0	0	0	0
GGA23	2	1	50	3	3
GGA24	2	1	50	13	13
GGA25	2	0	0	0	0
GGA26	2	1	50	11	11
GGA27	2	1	50	1	1
GGA28	2	1	50	7	7
LGE22C19W28_E50C23	2	0	0	0	0
Sex chromosomes					
GGAW	1	1	100	15	15
GGAW_random^b^	1	0	0	0	0
GGAZ	6	5	83.3	17	3.4
GGAUn_random	1	1^a^	100	15	15

For footnotes, see Table 1.

### Cross-species hybridization

To evaluate efficiency of interspecies hybridization, we examined overgo positions relative to coding/non-coding regions of a gene/marker. Those include exons (coding sequence), 5' and 3' UTRs, and non-coding sequence (introns, intergenic regions) (data shown in additional file 1: Overgo Probes). We calculated the success rate of probes derived from the different types of sequence, i.e., coding and non-coding regions, and probe efficacy estimated as percentage of successful overgos is shown in Table 3. As expected, overgos derived from coding sequence demonstrated the greatest efficiency (~64%) in cross-species hybridization. Probes specific to 5' and 3' UTRs and those designed from introns and other non-coding sequence were considerably less effective (~14-16%). Several overgos matched exon-intron boundary regions, and their success rate was around 17%. If we take into account a total of 77 successful probes, their efficiency relative to overgo sequence type would follow a similar pattern (Table 3).

**Table 3:** The overall success rate of overgo probes and efficiency rate among 77 successful overgo probes

Overgo sequence type	No. of probes by type	No. of successful probes	Percentage relative to probes by type	Percentage relative to 77 successful probes
Coding regions	91	58	63.7%	75.3%
5' and 3' UTRs	37	6	16.2%	7.8%
Introns	27	4	14.8%	5.2%
Other non-coding regions	55	8	14.5%	10.4%
Exon-intron boundary	6	1	16.7%	1.3%

The data are specified depending on sequence type used for probe design

The overgos we used in the white-throated sparrow library screen came mostly from chicken sequences (N = 194). In addition, we used 19 turkey EST-based probes and only 3 zebra finch EST-based overgos. We estimated the success rate of cross-species hybridization according to the probe origin (Table 4). Additionally, we examined the distribution of successful probes by species and by sequence type (Table 4). Among a total of 65 successfully-hybridized chicken overgos, approximately 72% of probes were derived from coding sequence, while all turkey and zebra finch successful probes matched coding regions.

**Table 4:** Success rate of overgo probes in cross-species hybridizations

Interspecies hybridization	No. of probes by species	No. of successful probes	Percentage relative to probes by species	Overgo sequence type	No. of successful probes by type	Percentage relative to successful probes
Chicken-sparrow	194	65	33.5%	Chicken overgos		
				Coding regions	47	72.3%
				5' and 3' UTRs	6	9.2%
				Introns	4	6.1%
				Other non-coding regions	8	12.3%
Turkey-sparrow	19	9	47.4%	Turkey overgos		
				Coding regions	9	100%
Zebra finch-sparrow	3	3	100%	Zebra finch overgos		
				Coding regions	3	100%

The data are shown depending of species specificity of probes and efficiency rate of successful probes by species and by sequence type

### Chicken-passerine comparative map

**Figure 3:**
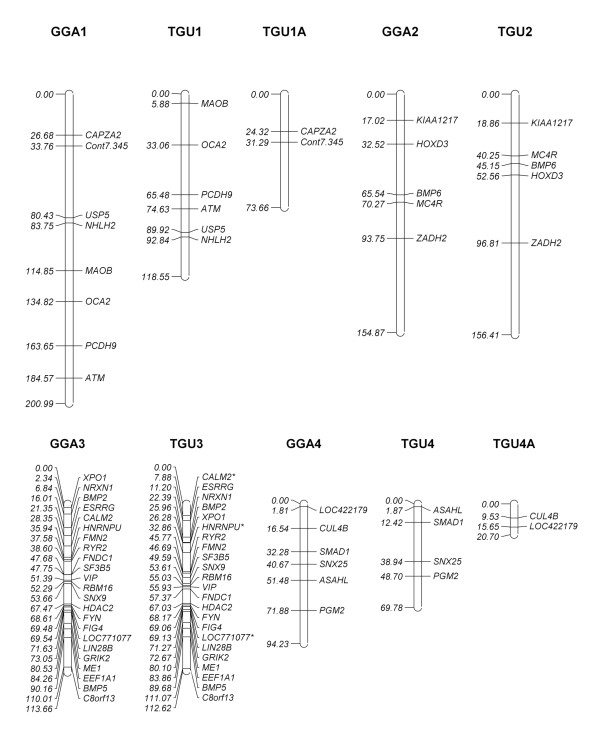
**The first-generation chicken-white-throated sparrow comparative cytogenetic map (chromosomes 1 through 4) based on sparrow BAC assignments**. The chicken chromosomes are designated as GGAn, their total lengths and loci positions being given in megabases. The orthologous zebra finch chromosomes (TGUn) are also shown. Gene/marker symbol marked with * means that an overgo sequence did not match any region in the zebra finch sequence according to Build 1.0, and the overgo coordinate is derived from the alignment of the appropriate chicken gene/marker sequence and the zebra finch whole genome sequence.

**Figure 4:**
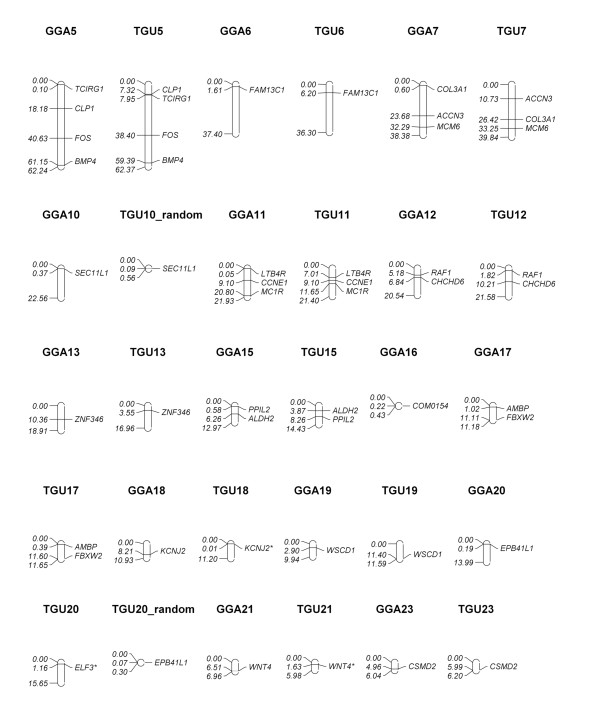
**The first-generation chicken-white-throated sparrow comparative cytogenetic map (chromosomes 5 through 23) based on sparrow BAC assignments**. Chicken chromosomes are designated as GGAn (total lengths and loci positions being given in megabases); orthologous zebra finch chromosomes (TGUn) are also shown. Gene/marker symbol marked with * indicates that an overgo did not match any region in the zebra finch sequence according to Build 1.0, and that the overgo coordinate is derived from the alignment of the appropriate chicken gene/marker sequence and the zebra finch whole genome sequence.

**Figure 5:**
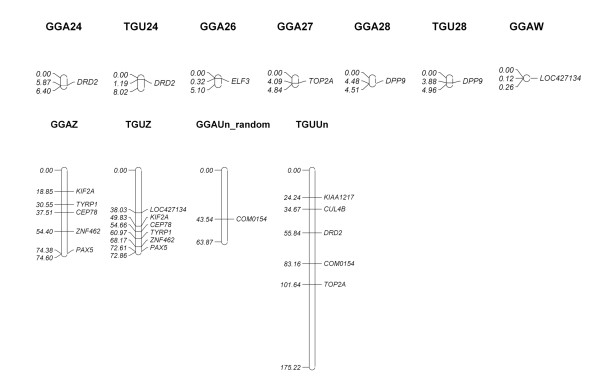
**The first-generation chicken-white-throated sparrow comparative cytogenetic map (chromosomes 24 through 28, Z, W, and random) based on sparrow BAC assignments**. Chicken chromosomes are designated as GGAn (total lengths and loci positions being given in megabases); orthologous zebra finch chromosomes (TGUn) are also shown. Gene/marker symbol marked with * indicates that an overgo did not match any region in the zebra finch sequence according to Build 1.0, and that the overgo coordinate is derived from the alignment of the appropriate chicken gene/marker sequence and the zebra finch whole genome sequence..

Based on the white-throated sparrow BAC library screen using the interspecies hybridization technique, we designed the first-generation chicken-sparrow comparative map (Figures 3, 4 and 5). The map embraced a total of 77 genes and markers on 26 chicken chromosomes including 24 autosomes and the two sex chromosomes, Z and W. The map for two chromosomes of particular interest, GGA3 and GGA4, contained respectively 23 and 6 genes/markers along their entire lengths. On three other macrochromosomes and the large Z chromosome, there are 4 to 8 genes/markers assigned per chromosome. We also mapped 1 to 3 BAC-gene assignments on three intermediate chromosomes and multiple microchromosomes as well as the W chromosome. Finally, we plotted on the map the appropriate zebra finch chromosomes that involve the same 77 loci. For a few loci, their exact position in the zebra finch genome remains unknown, and so they were arbitrarily placed on a non-specific chromosome, TGUUn.

## Discussion

Cross-species hybridization is efficient for deriving large-insert sequences for species whose genomes have not yet been fully characterized (e.g. [16,17,35]). With little to no sequence information, this comparative technique provides a starting point for genomic studies of non-model organisms. It is particularly useful for closely related species that exhibit much sequence homology and synteny. For example, the genomes of birds are relatively stable despite large evolutionary distances and an array of divergent phenotypes [39,40], so they lend themselves particularly well to comparative methodologies. Here we employed cross-species hybridization to identify relevant BAC sequences for the white-throated sparrow. By comparing identified markers across 26 chicken and zebra finch chromosomes, we developed a first-generation chicken-passerine comparative map showing BAC-gene assignments for the white-throated sparrow relative to the chicken and zebra finch chromosomal locations. Such information is a vital first step as it provides the framework for additional genome-wide studies. In addition, such comparisons create a basis for understanding how gene rearrangements affect the development and expression of complex phenotypic traits, and they form a foundation with which to study the evolutionary transitions that have occurred across taxa.

As expected, cross-species overgo hybridization proved more successful with probes derived from the coding regions of genes. In the present study, the overall efficiency of coding region probes was relatively high (64% compared to that for non-coding regions, which was 14-16%). Similarly, of the probes that successfully hybridized to white-throated sparrow BACs, approximately 77% of those were derived from the coding regions of genes. Therefore, by focusing efforts on coding areas within the genome, researchers can expect to increase their hybridization efficiency by 4 to 5 fold.

We also expected that probes derived from the more closely related zebra finch would bind more successfully than those from the more distantly related chicken and turkey, since the sequence divergence between galliform and passerine birds is much greater (about 100 million years) [41] than within the passerine lineage itself (approximately 24 million years) [42]. Although significantly fewer probes derived from the zebra finch were used in our study, all hybridized to white-throated sparrow BACs. As the zebra finch and other passerine genomes become more characterized, it will be possible to more fully test this hypothesis. Nonetheless, despite the evolutionary distances between chicken, turkey, zebra finch, and white-throated sparrow, cross-species overgo hybridization was still a highly effective technique.

Despite the relative evolutionary stasis among avian species [39], comparative mapping continues to also reveal significant differences [12,23,43]. In the white-throated sparrow, overgo hybridization efficiency seemed to differ according to chromosome (Table 2). For example, in three of the intermediate chromosomes (GGA6, GGA7, GGA8) and one macrochromosome (GGA5) each mapped with 4 overgos, hybridization success ranged from 0 to 100%. Amongst four macrochromosomes in which we used similar mapping effort (GGA1 through GGA4), hybridization success varied from 24 to 50%. Since we used overgos from both coding and non-coding regions, these differences could be due to a variety of factors including probe length, target length, temperature, genome duplications, as well as gene sequence homology. However, when mapping is confined to high-efficiency markers from coding regions, comparative cross-species overgo hybridization might reveal areas of differentiation. Since chromosomal rearrangements tend to occur in "fragile" regions of the genome [44], we would predict lower hybridization efficiency in such areas. Finally, since chicken microchromosomes have higher gene densities than macrochromosomes [8] and show higher recombination rates [45-47], we might expect relatively more overgo probes derived from coding regions to bind to microchromosomes.

Of a great interest in the white-throated sparrow is the identification and fine mapping of chromosomal rearrangements affecting morph-related genes. Thorneycroft [27,28] first showed that the association between morph and genotype (i.e., the presence or absence of ZAL2^m^) was absolute. Much later, researchers have shown that the rearrangement of ZAL2/ZAL2^m ^is complex, involving multiple inversions and perhaps linkages with other chromosomes [17,29,30]. However, it is still unclear how these rearrangements affect gene function in the two morphs. Characterizing the white-throated BAC library is an essential first step for physical and comparative mapping. Here we focused our study on a number of candidate genes that may be involved in regulating genetic differences between the white and tan morphs (see additional file 2: Targeted Candidate Genes for the list of candidate genes). Each of these genes plays an important role in controlling pathways with significant morphological and behavioral consequences. Importantly, some of them are located on GGA3 and GGA4 and might be directly affected with the intra-chromosomal rearrangements observed in the two morphs. In addition, positive white-throated sparrow BACs that were identified as certain genes or markers are being used in FISH to complete a cytogenetic map for both tan and white morphs of the white-throated sparrow. The FISH mapping will reveal new details about organization and evolution of ZAL2, ZAL3, and other chromosomes in this species and other related Emberizids. Eventually, it will be possible to identify sparrow clones that harbor affected candidate genes critical for regulation and manifestation of qualitative, reproductive and behavioral traits in two morphs. These BAC clones can be sequenced to reveal the DNA variation underlying the striking phenotypic differences between the two morphs. Importantly, BAC clones mapped to ZAL2/ZAL2^m ^and ZAL3/ZAL3^a ^may represent both normal and rearranged chromosomes because a single white female (ZAL2/ZAL2^m^) was used as a DNA source for the library construction [30]. This library can be used to reveal genomic differences (including breakpoints and affected genes) between the two morphs by BAC end sequence and FISH analyses (e. g. [18,48,49]).

A complete understanding of genomes will require both an interdisciplinary, systems-based approach [7,50] as well as a toolbase that extends far beyond model organisms [6]. Here we show the utility of cross-species overgo hybridization for characterizing BAC libraries of non-model organisms. Unlike other techniques that require sequence information (e.g. [51]), this technique can be accomplished with relatively little starting knowledge of the target genome. The result of such an analysis is a list of relevant genes and markers that can be used for physical mapping, linkage and candidate gene analyses. Furthermore, comparative overgo mapping advances our understanding of biological diversity by facilitating evolutionary comparisons across taxa that have diverged over 100 million years ago.

## Abbreviations

BAC: bacterial artificial chromosome; BLAST: Basic Local Alignment Search Tool; BLAT: BLAST-like Alignment Tool; CHORI: Children's Hospital Oakland Research Institute; EST: expressed sequence tag; FISH: fluorescence in situ hybridization; GGA: *Gallus gallus *chromosome; HDFR: High Density Filter Reader; NCBI: National Center for Biotechnology Information; TGU: *Taeniopygia guttata *chromosome; ZAL: *Zonotrichia albicollis *chromosome

## Competing interests

The authors declare that they have no competing interests.

## Authors' contributions

The project was elaborated and managed by EMT and RAG. MNR and JBD executed the hybridizations using overgo probes and equipment available in the JBD lab, and performed post-hybridization analyses. MNR plotted comparative chromosome maps and wrote up the manuscript draft. All authors contributed to the discussion of the results and preparation of the final manuscript version.

## Supplementary Material

Additional file 1 **Overgo Probes**. List and description of the 216 overgo probes used for the first screening of the white-throated sparrow BAC library. Click here for file

Additional file 2 **Targeted Candidate Genes**. Major candidate genes targeted in the first screening of the white-throated sparrow BAC library. Click here for file
