# Supplementary material for: Comparative BAC-based mapping in the white-throated sparrow, a novel behavioral genomics model, using interspecies overgo hybridization
Source: BMC Res Notes. 2011 Jun 21;4:211. doi: 10.1186/1756-0500-4-211 (PMC3155834; doi:10.1186/1756-0500-4-211)
Supplement: Additional file 1 — Overgo Probes. List and description of the 216 overgo probes used for the first screening of the white-throated sparrow BAC library. [file 1756-0500-4-211-S1.PDF]

Supplementary table 1: List and description of the 216 overgo probes used for the first screening of the white-throated sparrow BAC library.
Probes/genes are highlighted in different colors according to a species used as a sequence source for overgo design: yellow= chicken; tan=turkey; and gray= zebra finch.

Gene/contig symbol	Probe name (if different from gene/contig symbol)	Gene/marker name	GenBank Acc. No. of seqence used for overgo design	Chicken chromosome of BLAT/ BLAST match	Sequence origin	Probe location relative to nearest gene	Other notes	No. of positive sparrow BACs	Overgo start	Overgo end
LOC769217	***SHANK3***	hypothetical protein LOC769217	XM_001232454	chr1	CHICK GENOME	exon (2/3), intron (1/3)		0	362068	362105
CD36		CD36 molecule (thrombospondin receptor)	AJ719746	chr1	CHICK GENOME	coding region	also intron	0	12097269	12097314
CAPZA2		capping protein (actin filament) muscle Z-line, a	EH284752	chr1	TURKEY EST	coding region		8	26675724	26675763
Cont7.345	***CN229378***	RJB070F01.ab1 RJtestis Gallus gallus cDNA 5-	CN229378	chr1	CHICK EST	coding region?		4	33755800	33755840
EMP1		epithelial membrane protein 1	BX270611	chr1	CHICK GENOME	exon (50%), intron (50%)		0	50218955	50218998
CHUNK-1	***CHUNK1***	CHUNK-1 protein	AJ131560	chr1	CHICK GENOME	5' UTR		0	63114469	63114506
Contig4.721	***BU312632***	603543885F1 CSEQCHN61 Gallus gallus cDN/	BU312632	chr1	CHICK EST	coding region?		0	66775895	66775933
USP5		ubiquitin specific peptidase 5 (isopeptidase T)	BU104255	chr1	CHICK GENOME	coding region		11	80425069	80425108
HSD3B1	***HSD3B***	hydroxy-delta-5-steroid dehydrogenase, 3 beta- and steroid delta-isc		chr1	CHICK GENOME	intron	overgo match	0	81659138	81659402
NHLH2		nescient helix loop helix 2	AF123885	chr1	CHICK GENOME	intron	actual overgo	10	83747670	83747770
ARL6		ADP-ribosylation factor-like 6	AJ720909	chr1	CHICK GENOME	exon (85%), intron (15%)		0	94461204	94461241
ABCG1		ATP-binding cassette, sub-family G (WHITE), m	BX934614	chr1	CHICK GENOME	coding region		0	112744938	112744980
MAOB	***MAOA***	monoamine oxidase B	BU209877	chr1	CHICK GENOME	coding region	previously attr	6	114852543	114852580
PNPLA4		patatin-like phospholipase domain containing 4	CO773394	chr1	CHICK GENOME	NCR (5' end)		0	130115919	130115962
OCA2		oculocutaneous albinism II (pink-eye dilution ho	XM_425579	chr1	CHICK GENOME	coding region		3	134821808	134821848
Contig32.29	***BU210139***	604151264F1 CSEQCHN03 Gallus gallus cDN/	BU210139	chr1	CHICK EST	coding region?		0	145096759	145096802
DCT	***TYRP2***	dopachrome tautomerase (dopachrome delta-is	AF023471	chr1	CHICK GENOME	3' UTR or intron		0	150709017	150709054
PCDH9		protocadherin 9	BU379299	chr1	CHICK GENOME	coding region		14	163647492	163647529
DHRS12		dehydrogenase/reductase (SDR family) membe	EH288946	chr1	TURKEY EST	NCR (5' end)		0	174534969	174535004
ATM		ataxia telangiectasia mutated	AB026548	chr1	CHICK GENOME	coding region		2	184574221	184574259
UCP3	***UCP2***	uncoupling protein 3 (mitochondrial, proton carr	AF433170	chr1	CHICK GENOME	5' UTR		0	200547180	200547219
SEC11L1	***SPC18***	SEC11 homolog A (S. cerevisiae)	AW355399	chr10	CHICK GENOME	coding region		7	367720	367759
FBN1		fibrillin 1	U88872	chr10	CHICK GENOME	coding region		0	11742886	11742925
FURIN	***PACE***	furin (paired basic amino acid cleaving enzyme)	Z68093	chr10	CHICK GENOME	5' UTR (70%), exon (30%)		0	22277888	22277925
LTB4R	***lt4r1 (Salmo)***	leukotriene B4 receptor	XM_413985	chr11	CHICK GENOME	coding region		4	45827	45868
CCNE1		cyclin E1	U28981	chr11	CHICK GENOME	NCR (3' end)	also hits chrU	2	9101918	9101957
MC1R		melanocortin 1 receptor (alpha melanocyte stim	D78272	chr11	CHICK GENOME	coding region		30	20802862	20802900
RFT1		RFT1 homolog (S. cerevisiae)	AJ728632	chr12	CHICK GENOME	coding region		0	1134240	1134283
RAF1		v-raf-1 murine leukemia viral oncogene homolo	X07017	chr12	CHICK GENOME	coding region		3	5175628	5175667
CHCHD6		coiled-coil-helix-coiled-coil-helix domain contain	BX931932	chr12	CHICK GENOME	3' UTR		5	10095134	10095171
ENSGALG00000021557	***CN226563***	RJB083D06.ab1 RJtestis Gallus gallus cDNA 5-	CN226563	chr13	CHICK EST	coding region?		0	1049267	1049309
ZNF346		zinc finger protein 346	CN230815	chr13	CHICK GENOME	5' UTR	also hits chrU	6	10364795	10364836
UniGene Gga.15127	***CR385759***	Finished cDNA, clone ChEST690k11	CR385759	chr13	CHICK EST	coding region		0	18860998	18861038
ENSGALG00000003615	***BU261722***	603504203F1 CSEQCHN51 Gallus gallus cDN/	BU261722	chr14	CHICK EST	coding region		0	1598411	1598453
LYRM1		LYR motif containing 1	BX929957	chr14	CHICK GENOME	coding region		0	15688786	15688823
PPIL2		peptidylprolyl isomerase (cyclophilin)-like 2	EH284219	chr15	TURKEY EST	coding region		12	582246	582283
ALDH2	***ALDH1B1***	aldehyde dehydrogenase 2 family (mitochondria	BI066306	chr15	CHICK GENOME	coding region		5	6261165	6261202
LOC768783	***LEI0258***	hypothetical protein LOC768783	Z83781	chr16	CHICK GENOME	intron		0	101465	101504

GNB2L1		guanine nucleotide binding protein (G protein), l	CD765876	chr16	TURKEY EST	coding region		0	110935	110972
Contig4833.1	***COM0154***	Gallus gallus clone hm37155 genomic marker (	AF063648	chr16	CHICK EST	NCR	hits Un_rando	18	43539670	43539707
B-G	***MHCB***	MHC B-G antigen	M61864	chr16_random	CHICK GENOME	3' UTR	several other	0	183888	183931
AMBP		alpha-1-microglobulin/bikunin precursor	BG710015	chr17	CHICK GENOME	coding region		10	1024056	1024099
FBXW2		F-box and WD repeat domain containing 2	AJ452913	chr17	CHICK GENOME	coding region	also hits chrU	4	11111757	11111795
MYH2	***MYH@***	myosin, heavy chain 2, skeletal muscle, adult	J02714	chr18	CHICK GENOME	NCR (5' end)		0	378059	378098
KCNJ2		potassium inwardly-rectifying channel, subfamil	U20216	chr18	CHICK GENOME	5' UTR		18	8205174	8205216
CRK		v-crk sarcoma virus CT10 oncogene	L08168	chr19	CHICK GENOME	3' UTR		0	5144899	5144938
WSCD1		WSC domain containing 1	BU305563	chr19	CHICK GENOME	coding region		12	9638173	9638210
ASB10		ankyrin repeat and SOCS box-containing 10	BM488274	chr2	CHICK GENOME	coding region		0	163499	163536
VIPR1		vasoactive intestinal peptide receptor 1	AB029895	chr2	CHICK GENOME	3' UTR		0	1729098	1729137
KIAA1217		KIAA1217	CR523260	chr2	CHICK GENOME	coding region		4	17024749	17024787
HOXA3	***HOXD3***	homeobox A3	AF067959	chr2	CHICK GENOME	NCR (3' end)		5	32524493	32524536
GOLGA4		golgi autoantigen, golgin subfamily a, 4	BU425242	chr2	CHICK GENOME	coding region		0	49036906	49036947
EGFR		epidermal growth factor receptor (erythroblastic	M10066	chr2	CHICK GENOME	3' UTR		0	51966641	51966680
BMP6		bone morphogenetic protein 6	BM440114	chr2	CHICK GENOME	3' UTR		2	65542023	65542060
MC4R		melanocortin 4 receptor	AB012211	chr2	CHICK GENOME	NCR (5' end)		9	70268578	70268617
NR4A3		nuclear receptor subfamily 4, group A, member	BQ037755	chr2	CHICK GENOME	intron		0	91705617	91705656
ZADH2		zinc binding alcohol dehydrogenase, domain co	CD218970	chr2	CHICK GENOME	coding region		9	93745183	93745223
GDAP1		ganglioside-induced differentiation-associated p	BU275057	chr2	CHICK GENOME	coding region		0	123002877	123002914
LOC430962	***PLCG1***	hypothetical LOC430962	BU232536	chr2	CHICK GENOME	coding region	LOC419175 s	0	154506356	154506393
EPB41L1		erythrocyte membrane protein band 4.1-like 1	BU388686	chr20	CHICK GENOME	coding region		7	188729	188766
BMP7		bone morphogenetic protein 7	AF223970	chr20	CHICK GENOME	NCR (5' end)		0	11638489	11638528
SDF4	***TUS0022***	stromal cell derived factor 4	AI979776	chr21	CHICK GENOME	coding region		0	2529213	2529252
WNT4		wingless-type MMTV integration site family, mei	D31900	chr21	CHICK GENOME	coding region		7	6506125	6506162
DPYSL2	***CRMP2, CRMP1***	dihydropyrimidinase-like 2	U17277	chr22	CHICK GENOME	3' UTR	previously attr	0	464543	464582
NUDCD3		NudC domain containing 3	BX933007	chr22	CHICK GENOME	coding region		0	3934111	3934148
LOC419540	***ARID1A***	similar to AT rich interactive domain 1A (Swi1 lik	BU241617	chr23	CHICK GENOME	coding region		0	17639	17678
CSMD2		CUB and Sushi multiple domains 2	DN850390	chr23	CHICK GENOME	coding region		3	4959309	4959352
TNIP1	***ETS1***	TNFAIP3 interacting protein 1	S68254	chr24	CHICK GENOME	NCR (promoter)		0	937464	937503
DRD2		dopamine receptor D2	NM_001113290	chr24	CHICK GENOME	coding region		13	5874554	5874591
SMG5		Smg-5 homolog, nonsense mediated mRNA de	XM_423228	chr25	CHICK GENOME	NCR (3' end)		0	181	218
FCRL2	***FCR/L***	Fc receptor-like 2	BU232940	chr25	CHICK GENOME	intron		0	1709655	1709692
ELF3		E74-like factor 3 (ets domain transcription facto	BX932699	chr26	CHICK GENOME	coding region		11	317529	317567
NFASC	***NRF***	neurofascin	Y14347	chr26	CHICK GENOME	intron		0	1742343	1742382
LOC769232	***TRA@***	similar to T cell receptor alpha	U04611	chr27	CHICK GENOME	3' UTR		0	81417	81456
TOP2A		topoisomerase (DNA) II alpha 170kDa	AF285155	chr27	CHICK GENOME	coding region		1	4090618	4090661
MVP		major vault protein	AJ719400	chr28	CHICK GENOME	coding region	Jf_g1:76418-8	0	30242	30279
DPP9		dipeptidyl-peptidase 9	XM_001233752	chr28	CHICK GENOME	coding region	Jf_g2:361262	7	4479608	4479645
Contig72.23	***MCW0261***	MCW261 Chicken RPMACrooijmans Gallus gal	G32029	chr3	CHICK GENOME	NCR		0	800242	800279
XPO1		exportin 1 (CRM1 homolog, yeast)	AJ720676	chr3	CHICK GENOME	coding region		6	2344799	2344837
RTN4		reticulon 4	EH291226	chr3	TURKEY EST	intron		0	2458156	2458193
Contig66.104	***LEI0043***	G.gallus genomic DNA repeat region, clone 8F6	X78623	chr3	CHICK GENOME	NCR		0	3315905	3315944
OTOR		otoraplin	AF233518	chr3	CHICK GENOME	3' UTR		0	5589603	5589641
NRXN1	***ADL0177B***	neurexin 1	G01599	chr3	CHICK GENOME	intron		0	6841795	6841832
NRXN1	***ADL0177***	neurexin 1	G01599	chr3	CHICK GENOME	intron		12	6841948	6841985
LHCGR	***LHCGR (probe 2)***	luteinizing hormone/choriogonadotropin recepto	AY144675	chr3	TURKEY EST	intron		0	7531556	7531599

LHCGR	***LHCGR (probe 1)***	luteinizing hormone/choriogonadotropin recepto	AJ289775	chr3	CHICK GENOME	intron	0	7531731	7531770
JAG1		jagged 1 (Alagille syndrome)	X95283	chr3	CHICK GENOME	coding region	0	14431751	14431789
SNAP25		synaptosomal-associated protein, 25kDa	AH002473	chr3	CHICK GENOME	5' UTR or NCR (5' end)	0	14582288	14582326
BMP2		bone morphogenetic protein 2	BU423990	chr3	CHICK GENOME	NCR (3' end)	3	16010193	16010232
CAPN2		calpain 2, (m/II) large subunit	D38026	chr3	CHICK GENOME	3' UTR	0	18798241	18798280
TGFB2	***TGFB2 (probe 2)***	transforming growth factor, beta 2	AY298986	chr3	TURKEY EST	intron	0	20480088	20480128
TGFB2	***TGFB2 (probe 1)***	transforming growth factor, beta 2	X58071	chr3	CHICK GENOME	NCR (5' end)	0	20542237	20542376
ESRRG		estrogen-related receptor gamma	CD765669	chr3	TURKEY EST	coding region	1	21350074	21350113
LPGAT1	***ADL0370***	lysophosphatidylglycerol acyltransferase 1	U60789	chr3	CHICK GENOME	intron	0	23267735	23267774
CALM2	***CALM1***	calmodulin 2 (phosphorylase kinase, delta)	M31605	chr3	CHICK GENOME	NCR (5' end)	1	28350650	28350687
CAPN1	***CAPN1.5***	calpain 1, (mu/I) large subunit	X01415	chr3	CHICK GENOME	3' UTR	0	31551348	31551385
Contig9.294	***ADL0229B***	chicken STS ADL229, sequence tagged site	G01649	chr3	CHICK GENOME	NCR	0	33030550	33030583
Contig9.294	***ADL0229***	chicken STS ADL229, sequence tagged site	G01649	chr3	CHICK GENOME	NCR	0	33030571	33030609
SMYD3		SET and MYND domain containing 3	BX932267	chr3	CHICK GENOME	NCR (5' end)	0	35249363	35249404
HNRNPU	***GCT0011***	heterogeneous nuclear ribonucleoprotein U (sca	U62267	chr3	CHICK GENOME	intron	0	35883867	35883904
HNRNPU		heterogeneous nuclear ribonucleoprotein U (sca	EH286206	chr3	TURKEY EST	coding region	4	35941737	35941774
FMN2	***ADL0155***	formin 2	G01742	chr3	CHICK GENOME	intron	9	37580128	37580167
RYR2		ryanodine receptor 2 (cardiac)	BU362319	chr3	CHICK GENOME	coding region	7	38599675	38599714
ACTN2		actinin, alpha 2	X68797	chr3	CHICK GENOME	intron	0	39123610	39123649
LOC395933	***SULT***	sulfotransferase	AF033189	chr3	CHICK GENOME	3' UTR	0	43680659	43680698
T	***T (probe 2)***	T, brachyury homolog (mouse)	U25176	chr3	CHICK GENOME	3' UTR	0	44806943	44806982
PDE10A	***ADL0371***	phosphodiesterase 10A	U60781	chr3	CHICK GENOME	NCR (3' end)	0	45203635	45203674
PARK2	***LEI0115***	Parkinson disease (autosomal recessive, juveni	X85529	chr3	CHICK GENOME	intron	0	46656810	46656847
IGF2R		insulin-like growth factor 2 receptor	U35037	chr3	CHICK GENOME	3' UTR	0	47357856	47357895
FNDC1		fibronectin type III domain containing 1	CD765740	chr3	TURKEY EST	coding region	12	47679048	47679085
SF3B5		splicing factor 3b, subunit 5, 10kDa	XM_001232552	chr3	CHICK GENOME	coding region	4	47747965	47748002
C6orf72	***ABR0327***	chromosome 6 open reading frame 72	G16093	chr3	CHICK GENOME	intron	0	50030859	50030898
ESR1		estrogen receptor 1	U60211	chr3	CHICK GENOME	NCR (5' end)	0	50933303	50933340
C6orf98	***SYNE1***	chromosome 6 open reading frame 98	BU477376	chr3	CHICK GENOME	coding region	0	51192284	51192321
C6orf98	***MCW0187***	chromosome 6 open reading frame 98	L48899	chr3	CHICK GENOME	intron	0	51255329	51255363
VIP	***VIP (probe 2)***	vasoactive intestinal peptide	L36641	chr3	TURKEY EST	coding region	10	51391559	51391600
VIP	***VIP (probe 1)***	vasoactive intestinal peptide	X80905	chr3	CHICK GENOME	intron	0	51391623	51391662
RGS17	***MCW0004***	regulator of G-protein signaling 17	L40038	chr3	CHICK GENOME	intron	11	51592372	51592405
RBM16	***CF253873***	RNA binding motif protein 16	CF253873	chr3	CHICK GENOME	NCR (3' end)	0	52292798	52292836
SNX9		sorting nexin 9	CD765721	chr3	TURKEY EST	coding region	8	53658472	53658515
VTA1		Vps20-associated 1 homolog (S. cerevisiae)	EH286978	chr3	TURKEY EST	coding region	0	54947513	54947554
KIAA1244	***ADL0248 #1(B)***	KIAA1244	G01668	chr3	CHICK GENOME	exon (50%), intron (50%)	0	56336671	56336712
KIAA1244	***ADL0248 #2***	KIAA1244	G01668	chr3	CHICK GENOME	exon (50%), intron (50%)	0	56336671	56336712
MYB		v-myb myeloblastosis viral oncogene homolog (	X14612	chr3	CHICK GENOME	intron	0	57648427	57648466
Contig26.220	***ADL0280***	chicken STS ADL280, sequence tagged site	G01700	chr3	CHICK GENOME	NCR	0	57793484	57793523
Contig26.220	***ADL0280B***	chicken STS ADL280, sequence tagged site	G01700	chr3	CHICK GENOME	NCR	0	57793506	57793550
Contig1.187	***LEI0118***	Gallus gallus microsatellite DNA GgaMU145	X85540	chr3	CHICK GENOME	NCR	0	62732585	62732624
FABP7		fatty acid binding protein 7, brain	X65459	chr3	CHICK GENOME	coding region	0	63845094	63845133
Contig1.292	***MCW0277***	MCW277 Chicken RPMACrooijmans Gallus gal	G32038	chr3	CHICK GENOME	NCR	0	64302199	64302238
PLN	***PLN (probe 2)***	phospholamban	AH003051	chr3	CHICK GENOME	NCR (5' end)	0	65585517	65585556
PLN	***PLN (probe 1)***	phospholamban	AH003051	chr3	CHICK GENOME	NCR (5' end)	0	65585559	65585598
GOPC	***MCW0093***	golgi associated PDZ and coiled-coil motif conta	G31953	chr3	CHICK GENOME	NCR (3' end)	0	66048908	66048947
HDAC2		histone deacetylase 2	CD766014	chr3	TURKEY EST	coding region	16	67465362	67465405
FYN	***FYN (probe 2)***	FYN oncogene related to SRC, FGR, YES	X52841	chr3	CHICK GENOME	coding region	2	68609297	68609335

FIG4		FIG4 homolog (S. cerevisiae)	BU301538	chr3	CHICK GENOME	coding region		8	69484123	69484166
LOC771077	***AKD1, C6orf224***	hypothetical protein LOC771077	XM_001234378	chr3	CHICK GENOME	coding region		24	69541541	69541578
LIN28B	***MCW0252***	lin-28 homolog B (C. elegans)	G32020	chr3	CHICK GENOME	NCR (5' end)		2	71627923	71627961
Contig1.562	***ADL0115***	chicken STS ADL115, sequence tagged site	G01556	chr3	CHICK GENOME	NCR		0	72748863	72748902
GRIK2		glutamate receptor, ionotropic, kainate 2	XM_426186	chr3	CHICK GENOME	coding region		3	73051938	73051979
Contig1.603	***ROS0119***	Gallus gallus anonymous sequence from cosmi	AJ246049	chr3	CHICK GENOME	NCR		0	74065992	74066031
CCNC		cyclin C	U40873	chr3	CHICK GENOME	NCR (3' end)		0	74094994	74095034
COQ3		coenzyme Q3 homolog, methyltransferase (S. c	CD765538	chr3	TURKEY EST	coding region		0	74195133	74195172
Contig1.636	***ROS0108***	Gallus gallus anonymous sequence from Cosm	AJ231966	chr3	CHICK GENOME	NCR		0	75372139	75372178
Contig1.670	***GCT0019***	Gallus gallus microsatellite GCT19 AC repeat re	U97541	chr3	CHICK GENOME	NCR		0	76461927	76461966
CGA		glycoprotein hormones, alpha polypeptide	BI395075	chr3	CHICK GENOME	3' UTR		0	79320815	79320854
Contig1.753	***MCW0224***	MCW224 Chicken RPMACrooijmans Gallus gal	G31913	chr3	CHICK GENOME	NCR		0	79800394	79800433
ME1	***ME1 (probe 1)***	malic enzyme 1, NADP(+)-dependent, cytosolic	U49693	chr3	CHICK GENOME	NCR (5' end)		0	80487069	80487108
ME1	***ME1 (probe 2)***	malic enzyme 1, NADP(+)-dependent, cytosolic	AF408406	chr3	TURKEY EST	coding region		8	80532123	80532163
EEF1A1		eukaryotic translation elongation factor 1 alpha	L00677	chr3	CHICK GENOME	3' UTR		14	84257187	84257226
RIMS1	***ADL0306 #2***	regulating synaptic membrane exocytosis 1	G01721	chr3	CHICK GENOME	intron		0	84765856	84765895
RIMS1	***ADL0306 #1(B)***	regulating synaptic membrane exocytosis 1	G01721	chr3	CHICK GENOME	intron		0	84765856	84765907
Contig1.1000	***LEI0223***	G.gallus microsatellite DNA (LEI0223 (= MC19E	Z95313	chr3	CHICK GENOME	NCR		0	88147580	88147623
KHDRBS2	***UMA3.028***	KH domain containing, RNA binding, signal tran	G64875	chr3	CHICK GENOME	intron		0	88939334	88939371
BMP5		bone morphogenetic protein 5	S83278	chr3	CHICK GENOME	coding region		13	90161129	90161168
GSTA	***GSTA2***	glutathione S-transferase class-alpha	L15387	chr3	CHICK GENOME	coding region		0	91234224	91234261
Contig1.1157	***MCW0016***	Gallus Domesticus microsatellite marker MCW1	L40041	chr3	CHICK GENOME	NCR		0	93167342	93167380
Contig1.1183	***LEI0265***	G.gallus microsatellite DNA, LEI0265	Y10303	chr3	CHICK GENOME	NCR		0	94133779	94133818
Contig1.1278	***GCT0053***	Gallus gallus STS GCT053 (CA), sequence tag	AJ012245	chr3	CHICK GENOME	NCR		0	98112427	98112464
LOC422128	***MFAP1***	hypothetical LOC422128	M76679	chr3	CHICK GENOME	NCR (3' end)	true MFAP1 o	0	98153932	98153971
ODC1		ornithine decarboxylase 1	X64710	chr3	CHICK GENOME	3' UTR		0	99660303	99660342
MYCN		v-myc myelocytomatosis viral related oncogene	D90071	chr3	CHICK GENOME	intron		0	102116140	102116177
Contig17.414	***ADL0237***	chicken STS ADL237, sequence tagged site	G01657	chr3	CHICK GENOME	NCR		0	103804928	103804965
Contig17.338	***LEI0113***	G.gallus microsatellite DNA (53C2)	X82853	chr3	CHICK GENOME	NCR		0	106438881	106438920
RAB10	***GAR70.X2-6D***	RAB10, member RAS oncogene family	BF381430	chr3	CHICK GENOME	NCR (3' end)		0	107748675	107748698
SCARA5		scavenger receptor class A, member 5 (putative	CD765526	chr3	TURKEY EST	coding region		0	107944084	107944121
POMC		proopiomelanocortin (adrenocorticotropin/ beta-	AB019555	chr3	CHICK GENOME	NCR (3' end)		0	108173843	108173880
Contig17.151	***ADL0379***	Gallus gallus genomic sequence derived from L	AF315329	chr3	CHICK GENOME	NCR		0	109848297	109848336
C8orf13		chromosome 8 open reading frame 13	XM_001233099	chr3	CHICK GENOME	coding region		14	110013245	110013282
GAL8	***GAR83.C1-5B***	Gal 8	BF381443	chr3	CHICK GENOME	3' UTR (75%), exon (25%)		0	110240511	110240547
GAL2		gallinacin 2	AF033338	chr3	TURKEY EST	coding region		0	110257650	110257688
Contig17.74	***MCW0148***	MCW148 Chicken RPMACrooijmans Gallus gal	G31964	chr3	CHICK GENOME	NCR		0	112306048	112306085
GPR116		GPR116 G protein-coupled receptor 116	CO419996	chr3	CHICK GENOME	coding region		0	112717484	112717521
TNFRSF21		tumor necrosis factor receptor superfamily, men	AF349908	chr3	CHICK GENOME	3' UTR		0	112761536	112761574
MRPL19		mitochondrial ribosomal protein L19	BX930993	chr3	CHICK GENOME	coding region		0	113609951	113609990
LOC422179	***CN223064***	hypothetical LOC422179	CN223064	chr4	CHICK GENOME	coding region		25	1807532	1807569
HPRT1		hypoxanthine phosphoribosyltransferase 1 (Les	AJ132697	chr4	CHICK GENOME	3' UTR		0	4045553	404559
Contig11.449	***POU4F3***	G.gallus brn-3 gene (tissue type, liver)	X91997	chr4	CHICK GENOME	NCR	X91997 also h	0	11181089	11181122
CUL4B		cullin 4B	BM487435	chr4	CHICK GENOME	coding region		6	16538484	16538527
FGB		fibrinogen beta chain	M58514	chr4	CHICK GENOME	3' UTR		0	21397755	21397794
SMAD1	***MADH1***	SMAD family member 1	AF143239	chr4	CHICK GENOME	3' UTR		13	32283463	32283502
SNX25		sorting nexin 25	BU484876	chr4	CHICK GENOME	coding region		9	40674270	40674308
ASAHL		N-acylsphingosine amidohydrolase (acid ceram	BU399401	chr4	CHICK GENOME	coding region		16	51475867	51475906
CLOCK		clock homolog (mouse)	AF132531	chr4	CHICK GENOME	3' UTR		0	66978710	66978749

PGM2		phosphoglucomutase 2	BU252765	chr4	CHICK EST	coding region	also hits chrU	8	71875677	71875716
MSX1	***MSX1 (probe 1)***	msh homeobox 1	D10372	chr4	CHICK GENOME	3' UTR		0	81776979	81777016
Contig77.23, Contig77.24	***ALVEB4***	Gallus gallus sequence immediately downstreai	U54630	chr4	CHICK GENOME	NCR	also hits chr4:	0	91527529	91527568
TCIRG1		T-cell, immune regulator 1, ATPase, H+ transpo	AJ289021	chr5	CHICK GENOME	coding region		10	95745	95782
CLP1		CLP1, cleavage and polyadenylation factor I sul	AJ720420	chr5	CHICK GENOME	coding region		5	18182111	18182148
FOS		FBJ murine osteosarcoma viral oncogene homo	M18043	chr5	CHICK GENOME	NCR (5' end)		9	40632184	40632220
BMP4		bone morphogenetic protein 4	X75915	chr5	CHICK GENOME	coding region		10	61151284	61151323
FAM13C1		family with sequence similarity 13, member C1	BM426484	chr6	CHICK GENOME	coding region		8	1606967	1607006
COL13A1		collagen, type XIII, alpha 1	BU267500	chr6	CHICK GENOME	3' UTR		0	12148660	12148700
CYP17A1		cytochrome P450, family 17, subfamily A, polyp	M21406	chr6	CHICK GENOME	coding region		0	24818497	24818534
Contig73.62	***ALVE4***	Gallus gallus DNA, partial retroviral element ev	L40516	chr6	CHICK GENOME	NCR		0	35785665	35785704
COL3A1		collagen, type III, alpha 1	X00821	chr7	CHICK GENOME	coding region		9	601638	601675
NDUFS1		NADH dehydrogenase (ubiquinone) Fe-S protei	BU420938	chr7	CHICK GENOME	3' UTR		0	13658902	13658941
ACCN4	***ACCN3***	amiloride-sensitive cation channel 4, pituitary	XM_001232416	chr7	CHICK GENOME	coding region	previously attr	2	23675263	23675303
MCM6		minichromosome maintenance complex compo	G01587	chr7	CHICK GENOME	exon (75%), int	9 bp in intron	14	32286778	32286816
LOC424334	***Contig40.22***	hypothetical LOC424334	AADN02033762	chr8	CHICK GENOME	intron		0	576526	576567
PLA2G4A	***PLA2G2A***	phospholipase A2, group IVA (cytosolic, calciur	U10329	chr8	CHICK GENOME	5' UTR	previously attr	0	10016320	10016359
B4GALT2		UDP-Gal:betaGlcNAc beta 1,4- galactosyltransf	U19889	chr8	CHICK GENOME	3' UTR		0	21093622	21093659
FOXD3	***FOXD4***		U37274	chr8	CHICK GENOME	NCR (3' end)	previously attr	0	28563513	28563552
TF		transferrin	Y00407	chr9	CHICK GENOME	NCR (5' end)	overgo match	0	5643715	5644430
TFRC		transferrin receptor (p90, CD71)	X13753	chr9	CHICK GENOME	NCR (3' end)		0	16053897	16053934
ENSGALG00000009312	***EIF5A***	EST00362 Turkey Lambda ZAP Library Meleag	CD765676	chr9	TURKEY EST	coding region	overgo does r	0	21268904	21268945
CHAT1	***ADPRT***	ADP-ribosyltransferase	D31864	chrUn_random	CHICK GENOME	3' UTR or NCR	also hits chrU	0	17096114	17096153
LOC427134	***UBE2R2***	hypothetical LOC427134	BU122359	chrW	CHICK GENOME	coding region +	EST also hits	15	121354	121391
WPG	***CW01***	WPG pseudogene	D85614	chrW_random	CHICK GENOME	NCR		0	246216	246255
NOL6		nucleolar protein family 6 (RNA-associated)	BU216406	chrZ	CHICK GENOME	coding region		0	6798098	6798137
KIF2A	***KIF2***	kinesin heavy chain member 2A	CK311162	chrZ	ZEBRA FINCH EST	coding region		2	18854628	18854667
TYRP1		tyrosinase-related protein 1	AF003631	chrZ	CHICK GENOME	coding region		5	30553478	30553517
CEP78	***C9orf81***	centrosomal protein 78kDa	DV961380	chrZ	ZEBRA FINCH EST	coding region		1	37513465	37513502
ZNF462		zinc finger protein 462	CK307108	chrZ	ZEBRA FINCH EST	coding region		5	54404578	54404617
PAX5		paired box 5	AJ392389	chrZ	CHICK GENOME	coding region	also weaker to	4	74384656	74384695
SILV		silver homolog (mouse)	D88348	LGE22C19W28_	CHICK GENOME	3' UTR		0	489509	489546
GLI1	***GLI***	glioma-associated oncogene homolog 1 (zinc fi	U60762	LGE22C19W28_	CHICK GENOME	coding region	hits chrUn_ra	0	40848065	40848105
