# Supplementary material for: Comparative BAC-based mapping in the white-throated sparrow, a novel behavioral genomics model, using interspecies overgo hybridization
Source: BMC Res Notes. 2011 Jun 21;4:211. doi: 10.1186/1756-0500-4-211 (PMC3155834; doi:10.1186/1756-0500-4-211)
Supplement: Additional file 2 — Targeted Candidate Genes. Major candidate genes targeted in the first screening of the white-throated sparrow BAC library. [file 1756-0500-4-211-S2.DOCX]

Additional file 2: Targeted candidate genes.

Major candidate genes included in the first screen of the white-throated sparrow BAC library.

| **Gene name (synonym)** | **Symbol <alias>** | **Function** | **Chicken (GGA) and zebra finch (TGU) chromosome (Acc. No.)** | **GGA location, Mb** | **TGU location, Mb** |
| --- | --- | --- | --- | --- | --- |
| hydroxy-delta-5-steroid dehydrogenase, 3 beta- and steroid delta-isomerase 1 | HSD3B1 | metabolism, energy pathways; active steroid biosynthesis | GGA1 (NM_205118)  TGU1 (NM_001048264) | 81.7 | 90.8 |
| nescient helix loop helix 2 | NHLH2 | transcription factor activity, regulation of nucleobase, nucleoside, nucleotide and nucleic acid metabolism; sexual behavior, sperm development | GGA1 (NM_204797)  TGU1 (XM_002189168) | 83.7 | 92.8 |
| monoamine oxidase B | MAOB <MAOA> | oxidative deamination of biogenic and xenobiotic amines, metabolism of neuroactive and vasoactive amines in the central nervous sysytem and peripheral tissues, degrades amine neurotransmitters (dopamine, serotonin) | GGA1 (XM_416766)  TGU1 (XM_002190251) | 114.8 | 5.9 |
| dopachrome tautomerase (dopachrome delta-isomerase, tyrosine-related protein 2) | DCT <TYRP2> | melanin pigment biosynthesis; also regulates neural progenitor cell proliferation | GGA1 (NM_204935)  TGU1 (XM_002199349) | 150.7 | 41.7 |
| vasoactive intestinal peptide receptor 1 | VIPR1 | a receptor for vasoactive intestinal peptide, a small neuropeptide involved in smooth muscle relaxation and other metabolism | GGA2 (NM_204935)  TGU2 (XM_002193279) | 1.7 | 3.0 |
| protein KIAA1217 | KIAA1217 | affected by androgens | GGA2 (XM_418603)  TGU2 (XM_002190739; similar to sickle tail) | 16.9 | 18.8 |
| epidermal growth factor receptor (erythroblastic leukemia viral (v-erb-b) oncogene homolog, avian) | EGFR | cell proliferation; associated with fertility | GGA2 (NM_205497)  TGU2 (XM_002196432) | 52.0 | 32.3 |
| melanocortin 4 receptor | MC4R | interacts with adrenocorticotropic and MSH hormones and is mediated by G proteins; also associated with sexual activity and autosomal dominant obesity | GGA2 (NM_001031514)  TGU2 (XM_002199220) | 70.3 | 40.2 |
| nuclear receptor subfamily 4, group A, member 3 | NR4A3 | steroid-thyroid hormone-retinoid receptor, transcriptional activator; upregulated by estradiol | GGA2 (XM_419081)  TGU2 (XM_002193055) | 91.7 | 94.6 |
| bone morphogenetic protein 2 | BMP2 | member of the transforming growth factor-beta (TGFB) superfamily, induces bone and cartilage formation; also associated with spermatogenesis, and neural crest cell migration and ganglion formation in the enteric nervous system | GGA3 (NM_204358)  TGU3 (XR_054705) | 16.0 | 26.0 |
| estrogen-related receptor gamma | ESRRG | estrogen-related receptor and transcription factor | GGA3 (NM_001007081)  TGU3 (XM_002191088) | 21.4 | 11.2 |
| formin 2 | FMN2 <ADL0155> | cytoskeletal organization and/or establishment of cell polarity, associated with the formation of the meiotic spindle and dorso-ventral axis formation | GGA3 (XM_001235547)  TGU3 (XM_002192906) | 37.6 | 47.7 |
| estrogen receptor 1 | ESR1 | hormone binding, DNA binding, and activation of transcription; sexual development and reproductive function, sexual behavior | GGA3 (NM_205183)  TGU3 (NM_001076701) | 51.0 | 56.4 |
| vasoactive intestinal peptide | VIP | myocardial contractility, vasodilation, glycogenolysis, arterial blood pressure, relaxation of smooth muscle; territorial aggression | GGA3 (NM_205366)  TGU3 (XM_002187768) | 51.4 | 55.9 |
| dermatan sulfate epimerase | DSE | tumor-rejection antigen in the endoplasmic reticulum | GGA3 (XM_419777)  TGU3 (XM_002191471) | 66.5 | 66.0 |
| histone deacetylase 2 | HDAC2 | cell cycle, notch signaling; leads to the formation of heterochromatin and suppresses gene function | GGA3 (NM_204831)  TGU3 (XM_002192494) | 67.5 | 67.0 |
| glycoprotein hormones, alpha polypeptide | CGA | subunits of chorionic gonadotropin (CG), luteinizing hormone (LH), follicle stimulating hormone (FSH), and thyroid stimulating hormone (TSH) | GGA3 (XM_429886)  TGU3 (XM_002198276) | 79.3 | 78.9 |
| proopiomelanocortin | POMC | polypeptide hormone precursor for adrenocorticotrophin and lipotropin beta; pigment, stress, sexual activity, aggressiveness, metabolism | GGA3 (NM_001031098)  TGUUn (XM_002198019) | 108.2 | 0.002 |
| melanocortin 1 receptor (alpha melanocyte stimulating hormone receptor) | MC1R | melanogenesis (pheomelanin/eumelanin production), neuroactive ligand-receptor interaction | GGA11 (NM_001031462)  TGU11 (XM_002196038) | 20.8 | 11.6 |
| tyrosinase-related protein 1 | TYRP1 | melanogenesis, tyrosine metabolism, metabolic pathways | GGAZ (NM_205045)  TGUZ (XM_002193778) | 30.6 | 61.0 |
